# The Role of Stress in Breast Cancer Incidence: Risk Factors, Interventions, and Directions for the Future

**DOI:** 10.3390/ijerph18041871

**Published:** 2021-02-15

**Authors:** Deborah J. Bowen, Senaida Fernandez Poole, Mary White, Rodney Lyn, Debra A. Flores, Helen G. Haile, David R. Williams

**Affiliations:** 1 Department of Bioethics and Humanities, University of Washington, Seattle, WA 98195, USA; hghaile@uw.edu; 2 Office of the President, California Breast Cancer Research Program, University of California, Oakland, CA 94607, USA; senaida.poole@ucop.edu; 3 Atlanta, GA 30341, USA; whitemaryc@outlook.com; 4 School of Public Health, Georgia State University, Atlanta, GA 30302, USA; rlyn1@gsu.edu; 5 Kaiser Permanente Greater Southern Alameda Area, San Leandro, CA 94577, USA; debra.a.flores@kp.org; 6 Department of Social and Behavioral Sciences, Harvard University, Boston, MA 02138, USA; dwilliam@hsph.harvard.edu

**Keywords:** review paper, stress, psychological, breast cancer

## Abstract

Stress is a common belief among breast cancer patients and the public to explain variation in breast cancer incidence. Epidemiological studies interrogating the relationship between stress and cancer have reported mixed results. The impact of the topic and the lack of consensus has sparked this review of the literature to investigate gaps in knowledge and identify areas of research. We first present a brief summary of the biopsychosocial model generally used to conduct research on stress. We then divide the overview of the literature into areas of research focus. These include the role of distressing life events in breast cancer incidence, the role of adverse childhood events in later breast cancer incidence, the importance of race and socioeconomic status (SES) as social determinants of breast cancer incidence, and the specific role of chronic stress in relation to breast cancer. For each topic, we discuss the potential of stress as a risk factor and possible intervention strategies that could reduce the effects of stress. We then identify further research questions to be probed to fill the gaps in knowledge. We conclude with a discussion of future research directions for stress research as it relates to breast cancer incidence.

## 1. Introduction

Stress is a common idea in the general public to explain variation in breast cancer incidence. However, is stress related to breast cancer incidence, and, more importantly, can it be the target of an intervention to reduce breast cancer risk? This manuscript reviews evidence about the potential role of stress and identifies areas of future research activity to clarify and increase our understanding of stress in breast cancer.

We first present a brief summary of the biopsychosocial model generally used to conduct research on stress. Next, we divided the overview of the literature into distinct areas, each a focus of research attention. These include the role of distressing life events in breast cancer incidence, the role of adverse childhood events in later breast cancer incidence, the importance of race and discrimination and socioeconomic status (SES) as a social determinant of breast cancer incidence, and the specific role of chronic stress in relation to breast cancer. For each topic, we discuss the potential of stress as a risk factor and possible intervention strategies that could potentially reduce the effects of stress. We conclude with a discussion of future research directions for stress research as it relates to breast cancer incidence.

### Overview of the Biopsychosocial Model of the Stress Response

There is an emerging body of literature that puts forward the hypothesis that differential exposure to stressors and the co-occurrence of stressors may reflect part of the distinctive environmental contexts of demographic and exposure groups. As a result, these exposures contribute significantly to health disparities that have been observed in certain groups. We probe the existing literature on how to conceptualize and operationalize stress in order to examine how it affects health status. In turn, those exposures contribute in important ways to the observed patterns of disparities in health. The following text considers research on the conceptualization and operationalization of stress and how stressors can affect health status.

## 2. Psychosocial Stress

Stress is a nebulous concept that is subject to various interpretations. Stress is not a discrete value but rather a continuous interplay between an individual and their environment. Wheaton and Montazer [1] distinguished between three commonly used and interrelated terms in the stress process—stressors, stress, and distress. Stressors present as pressures in the environment, both internal and external, that create “conditions of threat, challenge, demands, or structural constraints that, by the very fact of their occurrence or existence, call into question the operating integrity of the organism.” These conditions of threat can manifest in the form of idiosyncratic events (e.g., job loss, divorce, or assault) to persistent stressors (e.g., food insecurity, poverty, rush hour traffic, or other exacerbating circumstances). Marginalized groups, such as people of color and those who are sexual minority persons, may experience an additional layer of stress due to discrimination, the threat of violence, and a higher likelihood of many discrete and chronic stressors being present at one time [2].

However, it is important to note that not every individual experience induces stress in the same way. The individual’s personal and social health stress (e.g., social support structures and access to medical and psychological care), in addition to the severity of the stressor, affect the ability of the individual to cope with the resulting stress. Stress, described as “a biological state of the body—a generalized physiological alert—in response to threatening agents,” [3] is often referred to as the “fight-or-flight” response. That is, a stressor is as much a physical response to an individual’s environment as it is psychological. It may cause hormonal, neurological, and/or cardiovascular changes in the body in order to compensate for the stressor. The nature of the stressor, and its frequency and severity, affect the psychological and biological response to particular stressors. In the mathematical field, to apply stress on an object means to apply “an external force acting against a resisting body.” However, the object applying force is not characterized as stress until the force of the object exceeds the “elastic limit” of the object being stressed upon. It is at this point that stress occurs, i.e., when the integrity of the body being acted upon cannot be maintained [4]. Pearlin [5] theorized that psychosocial stress on the human body acts in a similar fashion. When drawing his comparison, he separated the concept of stress from that of strain. Stress occurs when an individual perceives an event or environmental change as exceeding their capacity to adapt. In contrast, the concept of strain is similar to that of allostatic load [6]. Strain occurs when the individual reaches “the ultimate elastic limit.” At this point, the individual is no longer able to respond adaptively to environmental demands. The concept highlights the idea that disparate demands on the body can produce disparate evidence of physiological dysregulation [4].

The ability to access resources that have the ability to meet the demands of the stressor greatly alters the response, both psychologically and physically, to the stressor(s). This governs whether a particular stressor will cause a state of distress, commonly characterized as “stress.” For some, an amplified physiological response has acute and long-term consequences (e.g., inflammation, cardiovascular damage, and hyperventilation). Portions of the brain that are central to emotional and stress-related processes, such as the amygdala, also contribute to the mediation of stress-induced reactions linked to physical health outcomes. Studies have shown that differences in amygdala reactivity are associated with stressor-induced changes in blood pressure and cardiovascular disease risk, and increased secretion of the stress hormone, cortisol [6]. For others, psychosocial distress can lead to maladaptation in the neurological system, as biological stress coping can lead to a sustained allostatic dysregulation under chronically stressful conditions (allostatic load) [7]. This can cause the development or proliferation of harmful coping activities, such as tobacco and/or substance use, which can propagate negative mental and physical health effects. Likewise, an individual who has adapted to chronic stress may still encounter mental health effects of stressors without physiological evidence of them [7]. Regardless of how the stressor affects the health of the individual, the stress processes reflect an important pathway through which the social environment can “get under the skin” and have biological consequences.

## 3. Overview of Stress

### 3.1. Introduction

Early stress research focused on the biological processes through which stress affects individuals. There was no particular focus on distinguishing between various types of stressors, although there were considerations of different, both internal and external, factors that may alleviate or amplify the impact of the stressors on an individual. An example used by Zinzi and Williams highlighted this concept by noting that “the average person would be alarmed by close proximity to a lion, activating stress hormones such as epinephrine. However, for a lion tamer, this would be part of a daily routine and he or she could have the psychological and physical resources to draw on that would minimize any potential negative effects” [4]. While most people do not have jobs that expose them to dangerous or life-threatening occupational hazards, there are disparate experiences when it comes to other stressors such as pollution, traffic, technology, or prejudice. Early literature argued that the physiological response to stress is the same regardless of its source, as the body will enter a heightened state of alert (fight or flight) in order to meet the demands of the stressor. While the intensity may vary, a charging lion and a traffic jam both produce a stress response.

The exhaustion stage of Selye’s generalized adaptation syndrome describes what occurs when a chronically stressed individual is not given adequate time to “repair” or recover. In order to cope with the chronic exposure to the stressor in the absence of any respite, the body will raise its homeostatic set point for catecholamine (hormones made by the adrenal glands such as epinephrine, norepinephrine, and dopamine) levels, and that of other hormones, so the body can continue to response without restocking its stores. This leads to unwanted consequences, such as the body being unable to repair and recover itself, or fight off external infection. The exhaustion of the body’s resources can cause long-term weakening of numerous physiological systems via hormone dysregulation and physical damage to the nervous system. Additionally, extended exposure to certain external stressors (such as extreme heat or cold) can be fatal.

### 3.2. Current Views on Stress

Disparate stressors trigger disparate patterns of response from the body’s systems. The sympathetic–adrenal–medullary (SAM) system and the hypothalamic–pituitary–adrenocortical (HPA) axis, for example, are highly active in response to psychosocial stress. However, their activity levels vary depending on the source and type of stressor that prompted the response [4]. When the SAM system is activated, it causes the release of catecholamines, which work in tandem with the autonomic nervous system to modulate the cardiovascular, hepatic, pulmonary, musculoskeletal, and immune systems. They also produce mood changes [4]. The HPA axis, when activated, encourages the production of cortisol, otherwise known as the “stress hormone,” which aids in regulating numerous physiological processes (e.g., gluconeogenesis, anti-inflammatory responses, and fat and carbohydrate metabolism) and plays a role in causing compulsive overeating [4]. The SAM and HPA responses can be activated separately or together.

As noted in review articles, the perception that women under stress are more prone to cancer is common and long-standing [8,9]. In 1993, Hilakivi-Clarke et al. [10] put forward a conceptual model on the interactions between stress, personality, and psychosocial support on tumor growth based on evidence from human and animal studies. A similar multidisciplinary approach was used by Antoni et al. in 2006 [11] and Antonova et al. in 2011 [12] to develop proposed causal mechanisms for stress and breast cancer. We summarize here across these reviews.

Stressful life events trigger psychological processes that can affect health behaviors and neuroendocrine regulation through four main pathways—(1) stressors can lead to symptoms of distress and increase risk of mental disorders, adversely affecting psychological well-being due to the negative emotions associated with stress, (2) the negative emotional responses lead to biological dysregulation that can contribute to indicators of subclinical disease, (3) attempts to cope with negative emotional responses can lead to an increase in risky health behaviors (tobacco, other substances, over or under eating), including (4) a reduction in healthcare-seeking behavior (e.g., utilization of and engagement of health care services).

Studies from molecular biology have revealed the physiologic effects of the stress hormone cortisol on mammary gland development, estrogen activity, and other intra-cellular pathways involved in breast cancer [12]. Over time, the scientific evidence has strengthened regarding the biological plausibility that stressful life events lead to an increased chance of developing breast cancer in women.

#### Life Events

Stressful life events are discrete, psychosocial events that disrupt normal life. Stressful life events can be positive or negative, but most studies focus on negative or adverse life events. For example, stressful life events can include the death of a spouse, close relative or friend, end of a relationship (e.g., divorce/separation), personal illness or injury, loss of a job or change (e.g., a change to a more difficult kind of work), change in the health of a family member, and/or the gain of a family member [13]. Different measures have been used by researchers to assess stressful life events, including standard psychometric inventories (e.g., Holmes and Rahe scale, Brown–Harris schedule) and study-specific questionnaires [14,15].

An extensive body of epidemiologic studies has interrogated the relationship between stressful life events and breast cancer incidence. This literature has been the subject of multiple systematic reviews and meta-analyses [16,17,18,19]. This body of epidemiologic research reflects a variety of methodologic choices, each with its own potential biases, making it difficult to summarize results across studies or draw meaningful inferences. As highlighted by Burtow [9], the heterogeneity in methods reflect the need for distinct hypotheses based on a theoretical causal model. Another area examines the relationship between stressful life events and cancer. Increasingly, evidence suggests that stressful life events may be connected to an increased risk of breast cancer [13,16,20,21,22,23,24,25]. A systematic review and meta-analysis of cohort studies [16] reported that a history of stressful life events might slightly increase breast cancer risk (RR = 1.11 (95% confidence interval (CI) 1.03–1.19)). Similarly, Lin [20] conducted a meta-analysis including seven case-control or cohort studies, including 99,807 women, to investigate the association between striking life events (characterized by aversive anguishing experiences) and primary breast cancer susceptibility. It found that women with striking life events were at 1.5-fold greater risk of developing breast cancer (95% CI 1.15–1.97, *p* = 0.003). The pooled odds ratio (OR) for severe striking life events and breast cancer was 2.07 (95% CI 1.06–4.03), suggesting that women that experienced severe striking life events were at two-fold greater risk of developing breast cancer. Other studies have found no link between stressful life events and breast cancer incidence [26,27,28,29,30] resulting in contradictory literature on this topic.

A critical issue relates to timing, both in terms of latency and windows of breast susceptibility over the lifespan [12]. Investigators varied in their choice of study design, comparison group, time frames, measures of stressful life events, and control of confounders. All reviews reported inconsistent or conflicting results across multiple studies, which could be attributed to differences in study methods and misclassification error in measures of stress, confounders, and outcomes.

Petticrew et al. [29] performed a systematic review of 29 studies published prior to 1999 and concluded that the highest quality studies did not support a causal relationship between breast cancer and adverse life events. Butow et al. [9] examined 16 studies that met minimum quality standards, published between 1975 and 1996. These authors concluded that the evidence linking breast cancer incidence with adverse life events was weak and that the strongest predictor was severely threatening life events, such as the loss of a loved one, inferencing the possibility of a threshold of the stress response. They also concluded that the possibility of an association between breast cancer and adverse life events could not be ruled out. Duijts et al. [30] conducted a meta-analysis of the relationship between stressful life events and breast cancer risk based on 27 studies published between 1966 and 2002. They reported summary odds ratios for different categories of stressful life events. After adjusting for publication bias, these authors concluded that only the death of a spouse was associated, modestly, with breast cancer risk.

Bahri et al. [16] performed a systematic review of 11 cohort studies published between 2000 and 2016. These authors found that a slightly elevated pooled risk ratio was associated with a history of stressful life events and breast cancer. These authors recommended more research on perceived stress and the use of coping skills. They also advised psychological and counseling services be considered for women who experience stressful life events.

A recent study by Fischer et al. [31], not included in the previous review articles, examined the association of breast cancer risk and life events perceived to be stressful. These researchers hypothesized that the perception of stress from major life events would increase cortisol signaling, impair immune surveillance and increase breast cancer risk. Using a case-control design, they identified a significant, dose-response relationship between a cumulative measure of perceived stressful and adverse life events and an increased risk of breast cancer. Based on their analysis, they suggested that stressful life events have a greater impact on pre/peri-menopausal than post-menopausal cancer risk. The authors recommended further research on the combined effect of life events and coping style and social support, citing an earlier case-control study by Price et al. [32] that had suggested an interactive effect between social support and highly threatening life events. This work highlights the larger issue of what Bruce Dohrenwend calls “intracategory variability” in the measures of stress [33]. That is, it may be that the current measures are not sensitive enough to capture whether a reported event is truly stressful. More research is needed here.

Given the complexity of breast cancer etiology, future studies on the significance of stressful life events would benefit from the combination of advances in molecular biology into study designs and the creation of transdisciplinary study teams. Investigators with the Nurse’s Health Study II began the Mind–Body Study (MBS), a nested study on 233 cohort participants, to understand the inter-relationships between various biologic measures and psychosocial factors previously identified as stress [34]. The investigators concluded that self-administration of biospecimens was feasible and that measures of psychosocial factors had moderate-to-high reproducibility. This suggests promising directions for future epidemiologic research on this issue.

Interventions—for women without breast cancer, several recent reviews have concluded that the evidence on mindfulness-based interventions (MBIs) is limited but suggestive of effects on biologic mechanisms thought to increase breast cancer risk [35,36,37]. Investigators at the University of California, Davis, and University of California, San Francisco recently reviewed the available literature on meditation and telomere biology [38]. Telomeres cap chromosomes and telomere length have been associated with age-related diseases, including cancer. They identified the need for more systematic research on meditation interventions and better measures of biological and cellular outcomes of these interventions. This paper focuses on portions of the literature on stress and the incidence of breast cancer, preventive interventions to reduce stress among survivors of breast cancer were beyond the scope of this review.

### 3.3. Adverse Childhood Events

#### Risk Factors

There are a sizable number of studies on the association between adverse childhood events (ACEs) and the risk of breast cancer in adulthood [39,40] ACEs have been linked with various stressors that are grouped into several categories, including psychological, sexual abuse, physical abuse, parental separation and household drug/alcohol abuse [29,41]. In fact, a recently released report on ACEs in California indicated that ACEs are linked to toxic stress and are amenable to interventions tailored to the life stage and cause of ACE [40]. This report further indicates that the effects of external stressors, such as the coronavirus disease (COVID-19) may be cumulative with the effects of ACEs, and that more research is needed to determine this. Several studies linked ACEs to other health outcomes that are linked to breast cancer such as alcoholism, tobacco use, and increased cortisol levels [29,31,32,39].

**Figure 1:**
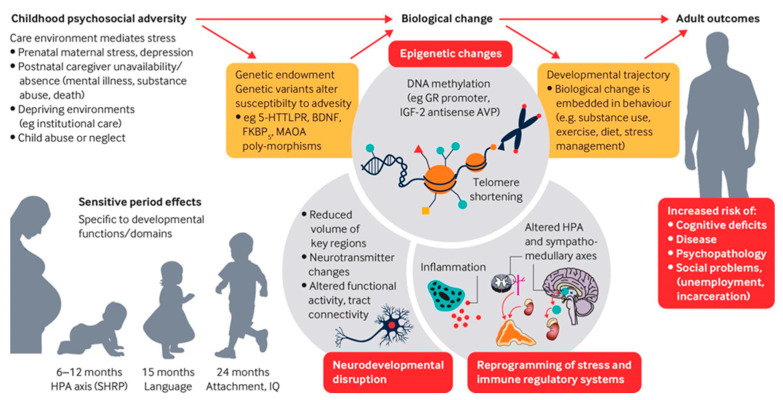
Childhood exposure to adverse childhood events (ACEs) and pathways to adult outcomes (from Nelson, et al. Adversity in childhood is linked to mental and physical health through life. *BMJ* **2020**, *371*, m3048. [43]. Shared under a creative commons open-access license.).

From a biological perspective, an abstract [42] focused on four biomarkers associated with breast cancer overall and with estrogen receptor-negative disease—adiponectin, C-peptide, high sensitivity C-reactive protein, and insulin-like growth factor-1. Based on this study, there appears to be a relationship between these biomarkers, ACEs, and increased risk of developing estrogen-receptor (ER)—breast cancer. These changes could have identified a mechanism for the ACE/stress effect, and indeed more broadly for the overall stress and negative disease outcomes. Figure 1 outlines the pathways to adult outcomes of childhood exposure to adverse events.

A challenge associated with the identification of ACEs is recall bias. The use of retrospective and prospective measures of adversity showed moderate agreement based on a comparative study conducted by Reuben et al. [12]. One study investigated the association between a history of social stress and breast cancer risk. A total of 11,467 women with no prior history of breast cancer participated in the prospective cohort study [3]. Summary measures of social adversity were delineated according to difficult circumstances in childhood, stressful life events, and perceived stress over a 10-year period. There were 313 incidents of breast cancer identified. There were no associations observed between any of the summary social adversity measures and subsequent breast cancer incidence, both with and without adjustment for age, menopausal status, parity, use of menopausal hormones, age at menarche, family history of breast cancer, etc.

The “Breakthrough Generations Study” was a prospective study of 106,612 women (median age = 46.6 years old) without a history of breast cancer in the United Kingdom, conducted between 2003 and 2012 [44]. The assessment tool included seven factors, and the women completed a follow-up survey every 2.5–3 years over a nine-year period. Of the 94.6% of the women who participated in the original study and completed a follow-up questionnaire, 1.7% developed breast cancer. In the follow-up, 34% of women described frequent or chronic stress, and 74% at least one adverse life event over the prior five years. Although it was shown that there was an inverse association with the death of a close relative and relative risk for breast cancer, overall, the study concluded that there was no statistically significant association between the frequency of stress or adverse life events during the five years prior to entry into the study and breast cancer risk [35]. The authors state that there was no sustaining evidence that self-reported stress and experience of adverse life events influenced the overall risk of breast cancer. However, their relatively sparse and uncited measures of stress indicate that perhaps there was no overarching model of stress that was used in the measurement selection. Failure to measure stress comprehensively can markedly understate the role of stress. Therefore, similar studies are needed that actually use a comprehensive measurement battery of stress responding in the cohort.

However, the authors of the study note “a lack of information on the intensity of stress, on stress in the workplace, and on the extent of social support or stress adaptive capacity” as a limitation in their study. A major finding in the stress literature is that failure to measure stress comprehensively markedly understates the role of stress. Hence, the key for future research is measures of cumulative stressors across multiple domains (e.g., with ACE being only one of them).

Interventions—it seems that we are in the early stages of discovery related to this topic and it is ripe for further research analysis. There are no published studies of interventions that ameliorate or reduce the effects of ACEs on stress reactions.

## 4. Racism as a Stressor

### 4.1. Introduction

The American Psychological Association has conducted surveys of communities across the United States since 2007 to provide a yearly snapshot of the experience of stress and its impact. The experience and impact of stress related to the COVID-19 pandemic cannot be overstated. When compared to white people, Black, Indigenous, and People of Color (BIPOC) experience more stress around meeting their basic needs (61% versus 47%), being able to access health care if they are sick (59% versus 46%), and contracting COVID-19 (71% versus 59%) [45]. While economics, political conflict, and racism have increased the amount of stress reported each year, the pandemic has created an amplified experience of stress across the nation. Additionally, most adults in the United States name police brutality toward Black, Indigenous and People of Color (BIPOC) as a significant stressor (59%). This is a 23% increase from 2016 (36%) when the question was added to the survey. Further, racism as a stressor was experienced in 2020 by a large proportion of Black (48%), Indigenous (42%), Latinx (42%), and Asian (41%) people.

**Figure 2:**
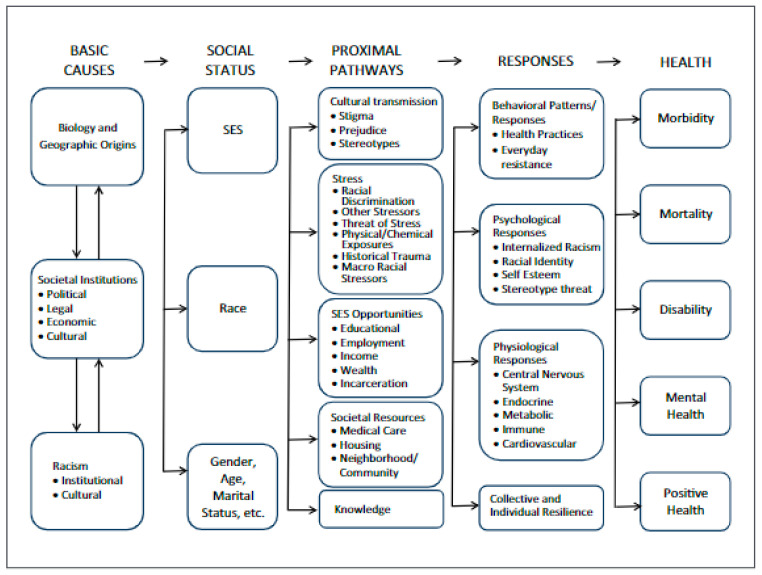
A framework for the study of racism and health (from Williams, 1997 [46]; Williams and Mohammed, 2013 [47]).

In introducing a framework for the study of racism and health, Williams [46] outlines multiple basic causes that act on and interact with social status factors, proximal pathways, and their responses to create and perpetuate health outcomes (see Figure 2). Notable aspects of the model are (1) fundamental determinants of health include institutional/systems and cultural racism; (2) as Williams described, these determinants are adaptable over time; and (3) by extension, interventions that focus solely on downstream proximal pathways (e.g., societal resources; stressors) without attending to systems and cultural level factors are unlikely to result in long term impacts on health disparities.

### 4.2. Risk Factors

Several systematic reviews, systematic meta-analyses, and longitudinal studies have examined racism as a risk factor for negative health outcomes and specific immunological responses. They have also investigated the association between racism and breast cancer incidence, and the relationship between immunological response and breast cancer incidence. Briefly, studies have demonstrated the impact of racism—on known risk factors for breast cancer such as drinking alcohol, on markers of inflammation, on mental health outcomes such as depression, and on physical health. Furthermore, examinations of longitudinal data have demonstrated links between the experience of racism and breast cancer.

#### 4.2.1. Markers of Inflammation

While academia has yet to reach a consensus on the role of inflammation in breast cancer (BC), several compelling investigations must be noted. Brody et al. [48] examined the relationships among experience of racism, racial identity, and cytokine levels (a marker of inflammation) in a longitudinal study of 160 African American 17–19-year-old adolescents, with a three-year follow-up when participants were 20–22 years old. Questionnaires were used to measure discrimination and racial identity, and blood was collected and used for measuring basal cytokine levels. At the three-year follow-up, investigators found main effects for discrimination (b = 0.307; *p* < 0.01) and racial identity (b = −0.179; *p* < 0.05) on cytokine levels. Furthermore, an interaction effect (b = −0.180; *p* < 0.05) was found, such that young people with negative racial identities who were exposed to high levels of racism at 17 had elevated cytokine levels three years later at 20 years old.

Moody et al. [49] examined the impact of everyday discrimination (e.g., frequency of racism in everyday life) on inflammation in racially diverse midlife women and found that higher everyday discrimination was linked to higher c-reactive protein over a seven-year period in non-obese women (body mass index (BMI) < 30).

While a 2009 meta-analysis concluded no significant association between C-reactive protein and breast cancer, Guo et al. noted high heterogeneity across studies (I^2^ = 51.0%) and a small number of breast cancer cases (*n* = 1024) suggest that the results should be interpreted with caution [50]. In contrast, a significant association was found in a systematic review that utilized a dataset with moderate heterogeneity (I^2^ = 45.9%), included 15 cohort and case-control studies representing international samples (six American studies, six European studies, and three Asian studies) and had 5286 cases of breast cancer. Change in C-reactive protein was related to breast cancer, with a combined OR of 1.16 (95% CI: 1.06–1.27) across the total sample. The association was stronger for Asian samples (OR = 1.57, 95% CI: 1.25–1.96) than for European (OR = 1.12, 95% CI: 1.02–1.23) and American (OR = 1.08, 95% CI: 1.01–1.16) samples [48].

#### 4.2.2. Mental and Physical Health Outcomes

Williams et al. [51], in reviewing 86 population-based empirical studies, noted significant associations between higher levels of perceived discrimination and both poor mental (e.g., depression and psychological distress), and physical health outcomes (e.g., well-being, self-rated health, and blood pressure). In addition, there were significant positive associations with risk factors for breast cancer (e.g., alcohol use). Greater exposure to racism was associated with greater alcohol consumption behaviors.

Similarly, in a 2006 systematic review of 138 quantitative papers examining self-reported racism and health, including outcomes that might be related to breast cancer [52], Paradies found a relationship between self-reported racism and poor health, with the strongest negative associations found for racism and mental health outcomes and health-related behaviors. Negative associations that were weaker but still significant were found between racism and physical health outcomes.

#### 4.2.3. Racism and BC incidence

Taylor et al. [53] examined the relationship between BC incidence and experience of everyday discrimination (e.g., frequency of racism in everyday life) and major experiences of racism (e.g., on the job, in housing, and interactions with the police) in a longitudinal sample of women from the Black Women’s Health Study. The investigators found significant relationships between major experiences of racism and BC incidence for younger women (<50 years old) in their sample of 593 BC cases. The incidence rate ratio was 1.32 (95% CI: 1.03–1.70) for discrimination on the job and the incidence rate ratio was 1.48 (95% CI: 1.01–2.16) for discrimination in all three areas (i.e., job, housing, and police).

Krieger et al. [54] examined the impact of legal racial discrimination (Jim Crow laws) on BC outcomes in US-born Black and white women. They found that the odds of being diagnosed with estrogen-receptor (ER-)negative BC were higher for Black women born before 1965 in a Jim Crow state, as opposed to those not born in a Jim Crow state (OR = 1.09; 95% CI: 1.06–1.13). However, Black women born after 1965 and white women born in any year did not experience this trend. If this relationship holds true, then these mechanisms could be valuable to evaluate in future research.

### 4.3. Interventions

Williams and Cooper [55] outline three strategies for systemic interventions on racism that will lead to reducing health disparities and describe interventions that have been effective within each strategy area.

The first strategy described is called “creating communities of opportunity,” which focuses on intervening within societal systems (housing, education, criminal justice) that prop up structural racism so that some of racism’s impacts on health are weakened. For example, high-quality interventions aimed at child development in children’s prenatal period and first five years of life have led to positive impacts throughout their lives. Notable outcomes include higher income for the intervention group compared to the control group, and more homeownership, lower rates of crime, fewer risky behaviors, higher education levels, lower Framingham risk score for coronary heart disease, less depression, and better blood pressure.

The second strategy is to “build more health into the delivery of medical care.” A subset of interventions in this area includes addressing the social needs of patients at the same time as the delivery of healthcare and diversifying the healthcare workforce. A 2020 initiative from the Office of the California Surgeon General on ACEs, toxic stress, and health [40] exemplifies this approach. In the “Roadmap for Resilience: The California Surgeon General’s Report on Adverse Childhood Experiences, Toxic Stress, and Health,” the authors propose an ACEs primary prevention strategy that relies on trauma-informed care within the healthcare setting and implemented throughout the state, coupled with effective collaboration between healthcare system, families, and external structural systems to facilitate and promote positive childhood experiences (PCEs).

The third strategy is “raising awareness of inequities and building political will to address them,” which describes several interventions that aim to shift attitudes, build awareness of racial health disparities, enhance empathy, and dismantle racism.

A key takeaway from their article is that there is a much larger body of evidence for effective interventions for racism than most scientists and lay audiences recognize.

## 5. Chronic Stress

### 5.1. Risk Factors

Many epidemiological and clinical studies have examined the interplay between stress, anxiety, depression, and breast cancer risk and progression [18,44,56,57,58,59,60,61,62]. The source of stress varies across the literature, including the loss of a loved one, divorce, stressful work environments, financial issues, personal injury, and other life events. Evidence to-date on the relationship between acute and chronic stressors and breast cancer risk is mixed. Some studies indicate a positive relationship between a history of stressful events and an increased risk of breast cancer [12,16,59,62,63]. In contrast, other studies have examined the relationship and found no relationship between stress and risk of breast cancer [44,56,57,58]. Researchers have attributed the variation in results to heterogeneous methodologies, recall bias, and measurement error.

Studies focused on job-related stress indicate that there is no appreciable increase in risk for breast cancer associated with stress in the workplace [51,56,58]. For example, a recent study investigated the effects of prolonged psychological stress in the workplace and the subsequent risk of cancer among women [56]. The study participants (*n* = 6571) were followed from January 2000 to December 2013, or until cancer diagnosis, emigration, or death. The results from this study revealed no significant relationship between job strain and an elevated risk of any form of cancer. Results from a separate study—a meta-analysis of prospective individual participant data from 12 European cohort studies—suggested that job strain was not linked with an elevated risk of any form of cancer (HR [hazard ratio] = 0.97, 95% CI 0.90–1.04; breast cancer HR = 0.97, 95% CI 0.82–1.14) [54].

In a recent prospective study, researchers observed the association between social support, life event stressors, and the risk of breast cancer development among women that are genetically predisposed to cancer for 15 years [57]. Previous acute and chronic stressors were assessed at baseline, and the women (*n* = 2739) responded to follow-up assessments in the following years. This study found no evidence that acute and chronic stressors played a role in increasing risk of breast cancer (acute stressors HR = 1.03 (0.99–1.08), *p* = 0.19; total chronic stressors HR = 1.0 (0.90–1.11), *p* = 0.98). The most recent systematic review on psychological stress and breast cancer incidence, which included 52 studies between 1966–2016, found that there is not enough evidence to state that chronic stress increases one’s risk of breast cancer [18]. Of the 52 articles included in their analysis, 26 articles positively linked stressful events and breast cancer, 18 articles rejected their hypothesis, and 8 remained unclassified. Findings were not conclusive, with authors noting that due to variations in study design, the types of psychological stress, methods of information gathering, and individual personality, they were unable to support or reject the possibility of an association.

The research on the relationship between stress and breast cancer is mixed, with evidence suggesting no association between job-related stress and breast cancer, evidence on overall stress seeming equivocal, and suggestions of a positive association between stressful life events and breast cancer. Still, findings across studies remain in conflict. The contradictory findings can be attributed to significant diversity in methodological approaches, including inadequate power and control of potential confounders, measurement error, and failure to consider potential moderators of stressor impact, such as stressor severity and chronicity, social support, personality, and coping style [57]. Determining the role of stress in the development of breast cancers is hampered by the complexity of isolating stress as a solitary variable [18]. There are a large number of risk factors that affect the severity of stress and its psychological impact, which activates the physiological response. These include access to social support, coping styles, health behaviors, and demographic variables such as age and income. Chirac [18] challenged researchers to recognize that “it is inaccurate to compare the death of a husband at a young age in a close family with a death at an old age, after a long period of disease or in a dysfunctional family.” The important takeaways here are that the field must come to recognize that it is not merely whether stressful experiences occur but how the stressful event is experienced and internalized by the individual that may matter most. Coping strategies and skills can serve as important mitigating factors in reducing the adverse consequences of stress [16]. Findings from the Chida [27] meta-analysis are relevant here because the study found that stressful life events do not affect cancer incidence but sensitivity to stress, unhealthy coping mechanisms, and negative emotional responses do. Thus, in future research, there is a need to assess and better understand stress adaptive capacity as a function of personality types, coping styles, and available familial and social support resources among individuals who serve as research participants.

### 5.2. Interventions

Given what is known about the relationship between stress and breast cancer, interventions targeting stress reduction are not guaranteed to decrease the risk of developing cancer. It is established in the literature that excess stress can have negative effects on human health. There is literature regarding several stress-reducing interventions such as mindfulness-based interventions (stress reduction and cognitive therapy), exercise, and social support. Social support (friends, romantic partners, and family) has been highlighted as particularly important in terms of perceived stress or psychological strains with an inverse association between the two being highlighted in many studies. [64,65,66,67,68,69]. Other studies have revealed exercise as a modest and temporary protective factor against acute psychological stress and occupational stress [70,71]. Lastly, researchers have reported reduced stress levels among participants of mindfulness-based stress reduction interventions [19,72,73,74,75].

## 6. Conclusions and Ideas for Future Research

We need to focus our research efforts on the places that will advance the field more rapidly and logically. For example, the best biologic and psychometric measures of psychosocial distress for community-based studies of stress and breast cancer risk, based on conceptual models of etiology and an assessment measure attribute, are not yet identified and used. This change could occur through the identification of the appropriate batteries of measures and models, and their use in large scale trials and observational studies of stress.

The batteries of measures and models should be developed, normed, and tested with the inclusion of multiple groups that experience significant health disparities, such as immigrants, disabled people, Black, Indigenous and People of Color (BIPOC), older adults, LGBTQ populations, poor people, etc. This process will allow the measures to detect and measure specific stress construct consistently across groups. Accuracy is critical for effective testing of models and testing of moderating and/or mediating relationships.

Together with this idea, we need to improve our research attention to the mechanisms of the development and maintenance of the stress response in multiple situations. This can only come about through the operationalization of conceptual models, the measurement of complete model variable sets, and the careful analyses of which variables are working in different situations. Some of the “outcomes” of stress reactions are ritually better conceptualized as possible mechanisms of stressful responding (e.g., health behavior change) and should be studied using mediational models to establish or refute their importance.

Specific findings of the role of ACEs in the development and maintenance of stress responding. Many concrete examples exist of questions t that need to be asked about the role of ACEs in stress. For example, do women with ACEs have more aggressive (triple negative, grade 3) breast cancer? Do identification and interventions regarding ACEs affect the response to treatment of breast cancer? Can interventions for reducing or eliminating reactions to ACE change the course of stress response?

The use of community-partnered participatory research methods is critical to building a nuanced picture of specific types of stress and their relationship with breast cancer. As noted earlier, there are disparate experiences of stress across ethnic groups, racism is a stressor that specifically impacts people of color groups, stress can act as a risk factor for behaviors that increase the likelihood of breast cancer incidence, and stress can produce changes in the body (e.g., inflammation) that may be linked with breast cancer incidence. Participatory research involves the building of authentic partnerships between the community and academic institutions; the equitable sharing of power, budget, and agenda-setting; both community and academia participating equitably in all phases of a research project, including dissemination; and co-ownership of data from the project. Partnering with the community can create a path to effective cancer interventions by centering the topics that are of interest to the community and incorporating community knowledge [76]. One example is partnering with the community to investigate factors that promote or inhibit breastfeeding among young mothers [77]. In this instance, the partnership surfaced specific barriers such as stigma that can impact breastfeeding of young mothers but may not be an issue for all groups of mothers equally. With participatory methods, the generalizability of knowledge from a project is improved because communities have partnered in designing the project.

## Abbreviations

SAM	sympathetic–adrenal–medullary system
HPA	hypothalamic–pituitary–adrenocortical
ACEs	adverse childhood events
COVID-19	coronavirus disease
SES	socioeconomic status
